# Dissociable dopaminergic and pavlovian influences in goal-trackers and sign-trackers on a model of compulsive checking in OCD

**DOI:** 10.1007/s00213-020-05636-3

**Published:** 2020-09-04

**Authors:** D. M. Eagle, C. Schepisi, S. Chugh, S. Desai, S. Y. S. Han, T. Huang, J. J. Lee, C. Sobala, W. Ye, A. L. Milton, T. W. Robbins

**Affiliations:** 1 Department of Psychology, University of Cambridge, Downing Site, Cambridge, CB2 3EB UK; 2 Sapienza University of Rome, Rome, Italy; 3 University of Bristol, Bristol, UK; 4 University College London, London, UK; 5 Oxford University Clinical Academic Graduate School, University of Oxford, Oxford, UK

**Keywords:** Obsessive-compulsive disorder, Sign-tracking, Goal-tracking, Quinpirole, Pavlovian, Rat

## Abstract

**Rationale:**

Checking is a functional behaviour that provides information to guide behaviour. However, in obsessive-compulsive disorder (OCD), checking may escalate to dysfunctional levels. The processes underpinning the transition from functional to dysfunctional checking are unclear but may be associated with individual differences that support the development of maladaptive behaviour. We examined one such predisposition, sign-tracking to a pavlovian conditioned stimulus, which we previously found associated with dysfunctional checking. How sign-tracking interacts with another treatment with emerging translational validity for OCD-like checking, chronic administration of the dopamine D_2_ receptor agonist quinpirole, is unknown.

**Objectives:**

We tested how functional and dysfunctional checking in the rat observing response task (ORT) was affected by chronic quinpirole administration in non-autoshaped controls and autoshaped animals classified as sign-trackers or goal-trackers.

**Methods:**

Sign-trackers or goal-trackers were trained on the ORT before the effects of chronic quinpirole administration on checking were assessed. Subsequently, the effects on checking of different behavioural challenges, including reward omission and the use of unpredictable reinforcement schedules, were tested.

**Results:**

Prior autoshaping increased checking. Sign-trackers and goal-trackers responded differently to quinpirole sensitization, reward omission and reinforcement uncertainty. Sign-trackers showed greater elevations in dysfunctional checking, particularly during uncertainty. By contrast, goal-trackers predominantly increased functional checking responses, possibly in response to reduced discrimination accuracy in the absence of cues signalling which lever was currently active.

**Conclusions:**

The results are discussed in terms of how pavlovian associations influence behaviour that becomes compulsive in OCD and how this may be dependent on striatal dopamine D_2_ receptors.

**Electronic supplementary material:**

The online version of this article (10.1007/s00213-020-05636-3) contains supplementary material, which is available to authorized users.

## Introduction

Checking is a functional response that provides information for guiding current behavioural choices. However, checking can become excessive or compulsive: self-reported checking is a significant predictor of OCD diagnosis compared with other OCD phenotypes (Stasik et al. 2012) and can become distressing and highly debilitating (Fontenelle et al. 2006; Grover et al. 2017; Zohar 1999).

It remains unknown whether excessive checking develops from functional checking or if they are separate behavioural phenomena regulated by different neural systems. Although excessive checking in OCD may arise due to obsessions that focus on threat and danger, the opposite has also been proposed—that excessive checking might drive the development of obsessions to rationalise or justify the compulsive action (Robbins et al. 2012). Thus, compulsive checking may initially arise, without pre-existing obsessional thought, from several neurobehavioural mechanisms, such as behavioural inflexibility, inability to terminate security-related behavioural patterns, or information-seeking, permitting investigation of the phenomenon using cross-species translational models (Hinds et al. 2012; Linkovski et al. 2013; Rotge et al. 2015).

Checking might provide information to decrease uncertainty, subsequently reducing anxiety in unpredictable circumstances. Supporting this is evidence that OCD patients, who are predominantly compulsive checkers, are more intolerant of uncertainty compared with other OCD subtypes (Rotge et al. 2015). However, it is also plausible that previous experience and/or individual differences shape future checking behaviour. Recent interest has focused on the relevance of motivational or emotional endophenotypes that bias future behavioural choices (Sarter and Phillips 2018). Of relevance to this study, the classification of individuals as sign-trackers or goal-trackers, with respect to pavlovian influences on behaviour (Flagel et al. 2007), might affect the importance of checking-related cues during checking behaviour (Vousden et al. 2020). Because sign-trackers and goal-trackers exhibit different, well-defined behavioural and neuropharmacological profiles (Clark et al. 2013; Cocker et al. 2016; Flagel et al. 2011; Flagel and Robinson 2017; Fraser et al. 2016; Lopez et al. 2015), they may respond differentially to pharmacological challenges that influence checking, helping to explain individual variability in these responses.

Growing evidence indicates that dopamine and the nucleus accumbens play significant roles in both OCD-relevant checking behaviour and sign-tracking/goal-tracking traits. In rodents, sensitization with the D_2_ dopamine receptor agonist quinpirole is widely used to generate compulsive-like behaviour (Amato et al. 2006; de Haas et al. 2011). Both chronic quinpirole administration and nucleus accumbens lesions increase checking in a manner superficially comparable with OCD compulsive checking (Ballester González et al. 2015; d’Angelo et al. 2017; Dvorkin et al. 2010; Eagle et al. 2014; Tucci et al. 2014). The dopaminergic dependence of checking appears to converge with the neuropsychopharmacological basis of sign-tracking, which is influenced by dopamine signalling in the nucleus accumbens core, with the suggestion that this is critical for the attribution of incentive salience during sign-tracking (Flagel et al. 2011). However, it remains unknown how, and if, sign-tracking and chronic quinpirole administration interact to produce additive effects on checking.

Here, we tested the hypothesis that excessive checking escalates from once functional behaviour if strong pavlovian-conditioned associations between checking and reward bias subsequent behavioural choices in favour of checking. Based on our recent work (Vousden et al. 2020), we predicted that the quinpirole sensitization model of checking and subsequent addition of uncertainty, both of which escalate checking in previous ORT studies (d’Angelo et al. 2017; Eagle et al. 2014), might differentially influence checking escalation in sign-tracker and goal-tracker rats. We show that the adaptive/maladaptive nature of checking can be explored using the ORT, reinforcing the potential of this task for direct translation between rodent and human studies (d’Angelo et al. 2017; Eagle et al. 2014; Morein-Zamir et al. 2018).

## Materials and methods

### Subjects

Male Lister-hooded rats (*n* = 48, Charles River, UK) were group-housed in fours and maintained at approximately 95% free-feeding weight. Experiments were conducted during the dark phase of a reversed 12-h light-dark cycle (lights off at 07:00). This research was conducted on UK Home Office Project Licence 70/7548 and was regulated under the Animals (Scientific Procedures) Act 1986 Amendment Regulations 2012 following ethical review by the University of Cambridge Animal Welfare and Ethical Review Body.

### Behavioural procedures

**Fig. 1:**
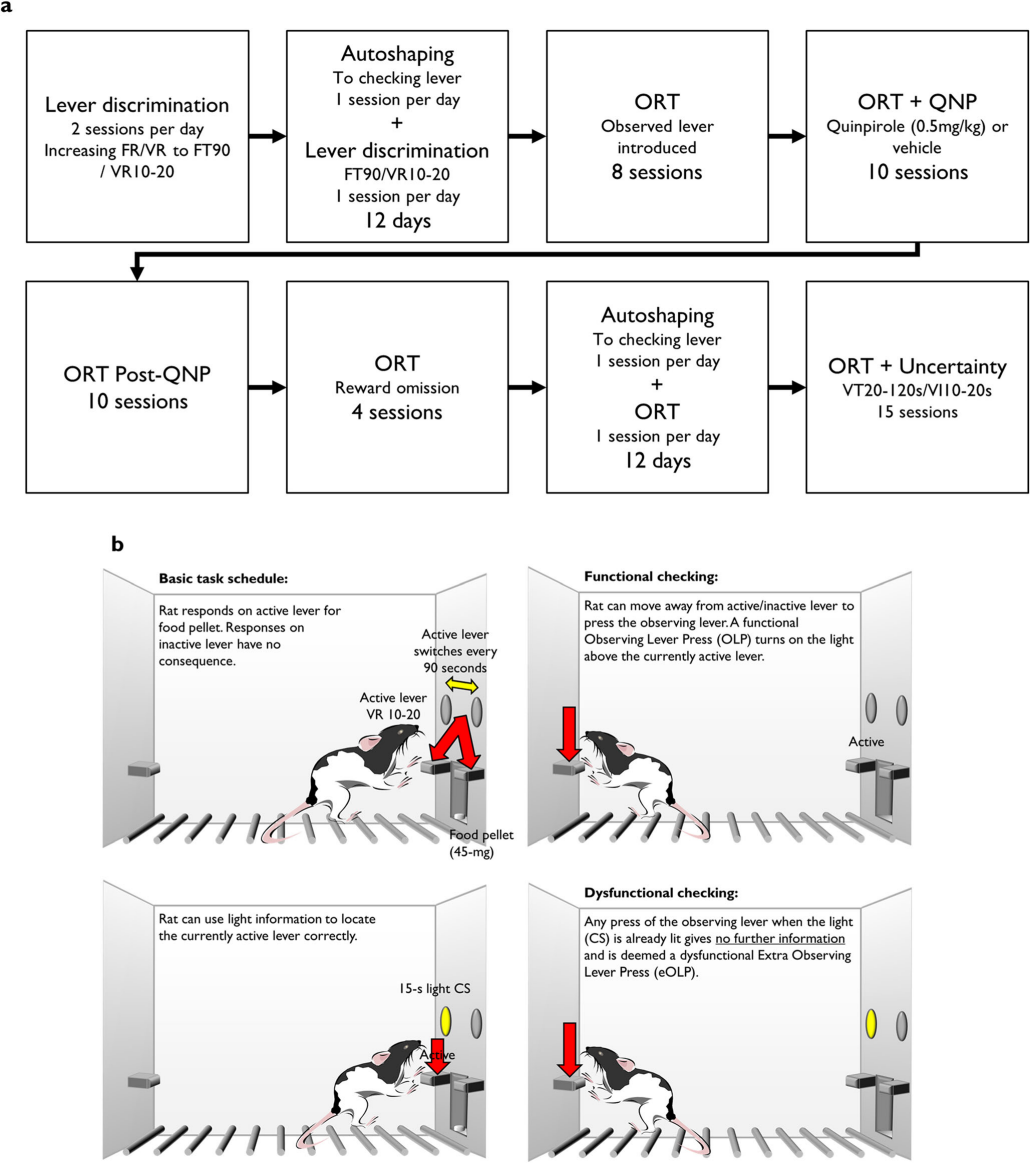
**a** Experimental timeline. **b** Schematic of the observing response task

A timeline of behavioural procedures is shown in Fig. 1a. Full details of the ORT apparatus and training procedures are described elsewhere (Eagle et al. 2014), and a simplified schematic of the task is presented in Fig. 1b.

#### Lever discrimination

Rats were trained in twelve operant conditioning chambers (Med Associates, Vermont, USA) to discriminate active (reinforced) and inactive (non-reinforced) levers on a variable ratio (VR10-20) schedule of reinforcement. Active and inactive levers switched position on a fixed time (FT90s) schedule. Rats initially received two training sessions per day. On the final 12 days of training, rats received one 32-min autoshaping session followed by one 21-min lever discrimination session, with rats returned to home cages for 1 h between sessions.

#### Autoshaping

The 48 rats were divided into two groups of 24, with half of the rats undergoing autoshaping and the other half serving as non-autoshaped controls. Both autoshaping and control sessions took place in the same chambers as lever discrimination training, but without the presentation of the active and inactive levers. Instead, rats that underwent autoshaping training were exposed to the lever that would subsequently be used as the observing lever, which was located on the opposite wall of the chamber to the location of the active and inactive levers during the lever discrimination sessions. Prior to autoshaping, rats had no experience of the observing lever.

Each session comprised a 2-min habituation period, followed by a 30-min autoshaping period in which rats received 30 10-s presentations of the lever on a FT30s schedule. Autoshaped rats received a pellet (45 mg Noyes Formula P pellets, Sandown Scientific, Middlesex, UK) immediately following lever presentation/retraction, while non-autoshaped control rats received the same number of pellets during the habituation period, but no pellets associated with lever presentation. Pellets were delivered into the magazine on the chamber wall opposite the observing lever.

#### Observing response task

Rats were trained in the same chambers previously used for lever discrimination. Each chamber had two retractable levers, with a light above each, to the left and right of a central magazine. As was the case during lever discrimination training, the active lever changed sides on an FT90s schedule, and active lever presses were reinforced on a VR10-20 schedule, as described previously (Eagle et al. 2014). Light illumination signalled that the lever below was currently ‘active’ (presses delivered reward pellets); the other lever was ‘inactive’ (presses had no consequence). Pressing the ‘observing’ lever on the back wall of the chamber illuminated the light above the active lever for 15 s and was recorded as a functional observing lever press (OLP). Pressing the observing lever while the light was illuminated had no further consequence and was recorded as dysfunctional extra observing lever presses (eOLPs). Rats were reinforced with a sucrose pellet for pressing the currently active lever, regardless of whether the light was illuminated. Chamber operation and online data collection were controlled with the observing response task program (written by A.C. Mar) for the Whisker server platform (Cardinal and Aitken 2010).

#### Assessing the effects of chronic quinpirole on ORT performance

Autoshaped and control rats were allocated to receive vehicle (VEH) or quinpirole (QNP), with groups matched for OLPs made on session 7 of ORT training, and autoshaped groups also matched for autoshaping performance. Rats received QNP (0.5 mg/kg, i.p.) or saline vehicle (1 ml/kg, i.p.) on 10 consecutive days, following the procedure described previously (Eagle et al. 2014). This single dose and regimen of quinpirole administration were used because it has previously been proposed as an animal model of OCD-like compulsive behaviour in rats (Szechtman et al. 1998; Winter et al. 2008). Briefly, on days 1–3, quinpirole treatment was given 60 min prior to testing to allow rats to overcome any immediate hypolocomotion or behavioural suppression associated with quinpirole administration. As behavioural suppression diminished across the first 3 days of treatment, on days 4–10, rats were administered with quinpirole 20 min prior to behavioural procedures.

#### Assessing the effects of reward omission on post-quinpirole ORT performance

The effect of reward omission on ORT performance was assessed in a single session in which no sucrose pellets were delivered, while all other task parameters remained the same. Performance on this session was compared with the baseline session that occurred the previous day, and two further sessions with the standard schedule of reinforcement, to assess any long-term effects of reward omission on checking.

#### Post-quinpirole autoshaping and ORT retraining

All rats received an additional 12 days of autoshaping (or control) training alongside ORT testing to rebaseline performance. During this phase of testing, rats received one autoshaping session, followed by one ORT session per day. The autoshaping and ORT sessions were separated by approximately 1 h, as were the behavioural sessions during the initial autoshaping phase of the experiment.

#### Assessing the effects of uncertainty on post-quinpirole ORT performance

Following a 30-day break from training, rats were tested on the ORT under conditions of uncertainty (see d’Angelo et al. 2017; Vousden et al. 2020, for full details).

Lever presses were reinforced on a variable interval (VI15s; range 10–20 s) schedule and active and inactive levers switched on a variable time (VT70s; range 20–120 s) schedule.

### Statistical analyses

For autoshaping data, approaches to the lever, total lever presses and total magazine entries were recorded during the CS lever presentation periods. As rats had received prior pellet reward training, magazine entries during autoshaping were higher than for conventional sign-tracking/goal-tracking studies, in which the rats are naive at the start of autoshaping. Therefore, group allocation was modified from Flagel et al. (2009) using the same procedures as Vousden et al. (2020). Briefly, allocation to sign-tracking and goal-tracking groups was based on the ratio of lever presses to magazine entries during the last two sessions of autoshaping training. Classification was conducted blind to ORT performance and was based upon clear splits in the distribution of the animals’ responding. For the ORT, results are expressed as rate (per minute) for comparability with human studies.

Behavioural data were subjected to full factorial ANOVA, with significance at *α* = 0.05. Homogeneity of variance was verified using Levene’s test. For repeated measures analyses, Mauchly’s test of sphericity was applied, and degrees of freedom were corrected using the Greenhouse-Geisser correction if ε < 0.75 and the Huynh-Feldt correction if ε > 0.75 for any terms involving factors in which the sphericity assumption was violated (Cardinal and Aitken 2006). ‘Pretraining’ (autoshaping vs. control) or ‘phenotype’ (sign-tracking vs. goal-tracking) and ‘drug’ (VEH vs. QNP) were between-subjects factors, and experimental block was a within-subjects factor. Additional within-subjects factors were included for analyses of active vs. inactive lever preference (‘lever’) and discrimination of the levers when the cue light was on or off (‘light’). Two rats were systematically excluded from analysis of ‘phenotype’ effects because they displayed an intermediate phenotype (see Supplementary Figure 1).

Omnibus ANOVAs were conducted to determine the effects of prior autoshaping, and its interaction with quinpirole treatment, on performance. Because of our *a priori* hypothesis that goal-trackers and sign-trackers would respond differently to quinpirole and behavioural challenges (reward omission and the use of uncertain reinforcement schedules), further analyses were conducted on the autoshaped animals alone, with Šidák-corrected pairwise comparisons being used to analyse specific interactions of interest. These analyses can be conducted to test *a priori* hypotheses even when the overall *F* test does not reach significance (Cardinal and Aitken 2006).

## Results

Key results are given below; additional results, including lack of baseline differences prior to each manipulation, are reported in Supplementary Materials.

### Classification of sign-trackers and goal-trackers

Over the course of training (Supplementary Figure 1), autoshaped rats approached the to-be observing lever (*F*_(3.35,154)_ = 4.05, *p* < .005, *η*^2^_*p*_ = 0.081), pressed the lever (*F*_(3.51,161)_ = 3.49, *p* < .012, *η*^2^_*p* =_0.071) and entered the food magazine (*F*_(2.70,124)_ = 3.48, *p* < .022, *η*^2^_*p* =_0.070), more than control rats, who received the same number of pellets in a manner explicitly unpaired with presentation of the to-be observing lever. Consistent with sign-tracking and goal-tracking classifications, across training sign-trackers approached (*F*_(3.32,66.3)_ = 4.77, *p* < .003, *η*^2^_*p*_ = 0.19) and pressed (*F*_(3.76,75.1)_ = 3.68, *p* < .01, *η*^2^_*p*_ = 0.16) the lever more than goal-trackers. However, there were no differences in magazine entries between sign-trackers and goal-trackers (session x group, *F*_(2.70,53.9)_ = 2.14, *p* = .112; group, *F*_(1,20)_ = 3.83, *p* = .064, *η*^2^_*p*_ = 0.16), likely due to the prior instrumental training. As expected, rats classified as sign-trackers showed a higher ratio of lever presses to magazine entries during the final 2 days of autoshaping (*F*_(1,21)_ = 5.53, *p* < .029, *η*^2^_*p*_ = 0.22).

### Quinpirole and autoshaping effects on functional and dysfunctional checking

#### Functional checking

**Fig. 2:**
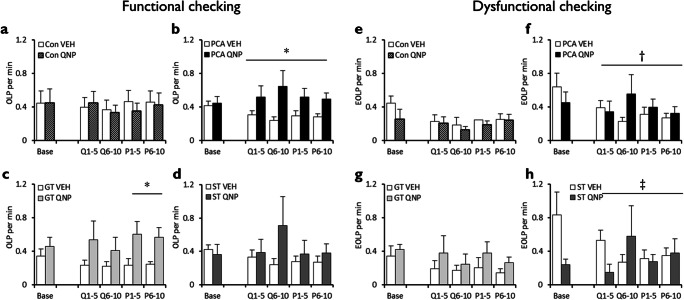
Effects of chronic quinpirole treatment on functional and dysfunctional checking on the observing response task (ORT). Chronic quinpirole did not affect functional checking in rats that had not undergone autoshaping (**a**) but increased functional checking in rats that had undergone prior autoshaping (**b**). Further analyses showed that the effects on functional checking were more pronounced and persistent in goal-trackers (**c**) than sign-trackers (**d**). Similarly, chronic quinpirole did not affect dysfunctional checking in rats that had not undergone autoshaping (**e**) but increased transiently dysfunctional checking in rats that had (**f**). ‘Base’ is baseline responding, Q1–5 and Q6–10 the first and second blocks of chronic VEH/QNP treatment; P1–5 and P6–10 the two post-treatment session blocks. Con, control group; PCA, autoshaped group; QNP, quinpirole-treated group; VEH, vehicle-treated group; GT, goal-trackers; ST, sign-trackers. Data are means ± s.e.m. Group sizes: Con VEH, *n* = 12; Con QNP, *n* = 12; PCA VEH, *n* = 12, PCA QNP, *n* = 12; GT VEH, *n* = 5; GT QNP, *n* = 6; ST VEH, *n* = 6; ST QNP, *n* = 5. Differences of *p* < .05 between VEH and QNP indicated by *; between non-autoshaped controls and autoshaped groups by †; between goal-trackers and sign-trackers, ‡

Quinpirole did not generally affect the number of functional OLPs made during or after chronic treatment (drug, *F* < 1; block x drug, *F* < 1), regardless of whether animals had undergone autoshaping or not (pretraining, *F* < 1; block x pretraining, *F* < 1; block x pretraining x drug, *F*_(2.09,91.9)_ = 1.74, *p* = 0.18). However, when non-autoshaped control and autoshaped rats were analysed separately, chronic quinpirole increased OLPs in autoshaped rats during and after the treatment period (Fig. 2b; drug, *F*_(1,22)_ = 4.29, *p* = .05, *η*^2^_*p*_ = 0.16; block x drug, *F*_(1.76,38.8)_ = 2.09, *p* = .14). By contrast, chronic quinpirole was found not to affect the number of functional OLPs in non-autoshaped controls (Fig. 2a; drug, *F* < 1; block x drug: *F* < 1).

Although an omnibus ANOVA did not show any differences between goal-trackers and sign-trackers in their OLP responses during and after chronic quinpirole treatment (phenotype, *F* < 1; drug x phenotype, *F* < 1; block x drug x phenotype, *F*_(2.03,36.5)_ = 1.65, *p* = .21), based on our *a priori* hypothesis that goal-trackers and sign-trackers would respond differently to quinpirole, we explored this further with Šidák-corrected planned comparisons (Cardinal and Aitken 2006). Further analysis of the autoshaped groups showed that chronic quinpirole increased functional OLPs selectively in goal-trackers (Fig. 2c) and only post-treatment (no differences between vehicle and quinpirole goal-tracking groups before or during treatment, all *p’s* > .18; quinpirole-treated goal-trackers made more OLPs post-treatment, all *p*’s < .043). There were no differences between vehicle- and quinpirole-treated sign-trackers (Fig. 2d) during or after chronic quinpirole treatment (all *p*’s > .094).

#### Dysfunctional checking

Quinpirole did not generally affect the number of dysfunctional eOLPs made during or after chronic treatment (drug, *F* < 1; block x drug, *F*_(3.10,136)_ = 2.17, *p* = .093, *η*^2^_*p*_ = 0.047), though autoshaped rats tended to make more eOLPs than non-autoshaped controls (Fig. 2d–e; *F*_(1,44)_ = 3.32, *p* = .075, *η*^2^_*p*_ = 0.07) irrespective of drug treatment (drug x pretraining, *F* < 1). Both goal-trackers (Fig. 2g) and sign-trackers (Fig. 2h) showed greater dysfunctional checking than non-autoshaped controls (phenotype, *F* < 1) with no overall interaction between quinpirole and sign-tracking/goal-tracking phenotype (drug x phenotype, *F*_(1,18)_ = 1.75, *p* = 0.20). However, there was a trend towards a differential effect of quinpirole on responding across blocks in goal-trackers and sign-trackers (block x drug x phenotype, *F*_(4,72)_ = 2.34, *p* = .063, *η*^2^_*p*_ = 0.12). Šidák-corrected pairwise comparisons revealed that this was due to a trend towards vehicle-treated sign-trackers showing greater baseline dysfunctional checking than vehicle-treated goal-trackers (*p* = .054). There were no other differences in responding across blocks between goal-trackers and sign-trackers treated with vehicle (all *p*’s > .12) or quinpirole (all *p*’s > .22).

### Quinpirole exerted an acute effect on lever pressing for reinforcement

Overall levels of active and inactive lever pressing on the ORT were reduced during quinpirole treatment (Supplementary Figure 2; block, *F*_(1.72,75.7)_ = 67.7, *p* < .001, *η*^2^_*p*_ = 0.61; block x drug, *F*_(1.72,75.7)_ = 60.1, *p* < .001, η^2^_*p*_ = 0.58). This suppression of responding was restricted to the quinpirole treatment period, as Šidák-corrected pairwise comparisons showed that there were no differences between vehicle- and quinpirole-treated groups at baseline (*p* = .28) or following quinpirole (*p*’s > .07), only during treatment (*p*’s < .001). Quinpirole suppressed responding on both active (*p*’s < .001) and inactive (*p*’s < .001) levers compared with baseline and post-quinpirole periods, though due to the higher baseline levels of responding, quinpirole more markedly suppressed active lever pressing (lever x block x drug, *F*_(2.11,92.6)_ = 26.1, *p* < .001, η^2^_*p*_ = 0.37). Rats that received quinpirole showed equal levels of responding on the active and inactive levers during the treatment period (all *p*’s > .74) but returned to pressing the active lever more after treatment (all *p*’s < .001). There were no differences in the effects of quinpirole on lever discrimination in goal-trackers or sign-trackers (lever x block x drug x phenotype, *F* < 1).

### Quinpirole acutely impaired the ability of rats to accurately discriminate between the levers in the absence of the cue light

**Fig. 3:**
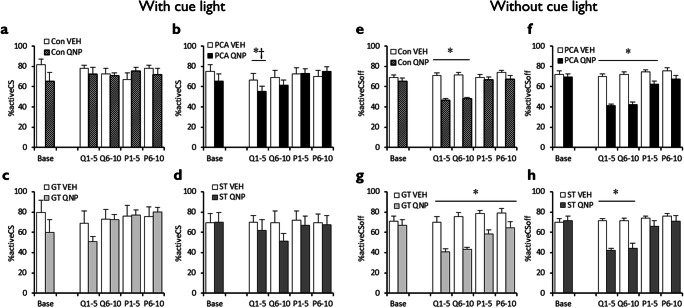
Effects of chronic quinpirole treatment on discrimination of the active and inactive levers in the presence and absence of the cue light identifying the active lever**.** Treatment with chronic quinpirole did not affect lever discrimination when cue light information was available in non-autoshaped controls (**a**) but acutely impaired discrimination in rats that had undergone prior autoshaping, albeit transiently (**b**). There were no differences in discrimination between goal-trackers (**c**) and sign-trackers (**d**). Quinpirole markedly impaired discrimination between the levers when the light was off, in both non-autoshaped control rats (**e**) and autoshaped rats (**f**). This effect was limited to the treatment period in control rats but persisted into the post-quinpirole period for autoshaped rats. The effect was also more pronounced in goal-trackers (**g**) than sign-trackers (**h**). ‘Base’ is baseline responding, Q1–5 and Q6–10 the first and second blocks of chronic VEH/QNP treatment, P1–5 and P6–10 the two post-treatment session blocks. Con, control group; PCA, autoshaped group; QNP, quinpirole-treated group; VEH, vehicle-treated group; GT, goal-trackers; ST, sign-trackers. Data are means ± s.e.m. Group sizes: Con VEH, *n* = 12; Con QNP, *n* = 12; PCA VEH, *n* = 12, PCA QNP, *n* = 12; GT VEH, *n* = 5; GT QNP, *n* = 6; ST VEH, *n* = 6; ST QNP, *n* = 5. Differences of *p* < .05 between VEH and QNP indicated by *; between non-autoshaped controls and autoshaped groups by †

On the ORT, rats can determine the currently reinforced, ‘active’ lever by making an observing lever press. As would be expected, rats were highly accurate in correctly choosing the active lever when the cue light was on (Fig. 3a–d; light, *F*_(1,44)_ = 7.56, *p* = .009, *η*^2^_*p*_ = 0.15). Vehicle-treated rats were able to maintain a high level of accuracy even in the absence of the cue light (Fig. 3e–h), but quinpirole-treated rats could not (drug, *F*_(1,44)_ = 11.7, *p* = .001, *η*^2^_*p*_ = 0.21; drug x light, *F*_(1,44)_ = 4.70, *p* = .036, *η*^2^_*p*_ = 0.10). This impairing effect of quinpirole differentially affected lever choice accuracy depending on whether the cue light was on or off (light x block x drug, *F*_(3.52,155)_ = 7.58, *p* < .001, *η*^2^_*p*_ = 0.15). Šidák-corrected pairwise comparisons showed that when the light was on (Fig. 3a, b) quinpirole only impaired lever choice accuracy early in the treatment period (Q1–5, *p* = .041), but not at any other time (all *p’s >* .075). When the light was off (Fig. 3e, f), quinpirole impaired lever choice accuracy both during quinpirole treatment (Q1–5 and Q6–10, all *p*’s < .001) and after quinpirole treatment (P1–5 and P6–10, all *p*’s < .024).

Lever choice accuracy also differed depending on whether animals had received prior autoshaping (Fig. 3b) or not (Fig. 3a; block x pretraining, *F*_(3.80,167)_ = 4.13, *p* = .004, *η*^2^_*p*_ = 0.086) as autoshaped animals showed reduced accuracy during the early quinpirole treatment period (Q1–5, *p* = .002) but not at any other point during testing (all *p*’s > .76). However, further analyses showed that there were no differences in the lever choice accuracy of goal-trackers (Fig. 3c) or sign-trackers (Fig. 3d), with all animals being equivalently affected by quinpirole administration (phenotype, *F* < 1; drug: *F*_(1,18)_ = 6.03, *p* = .024, *η*^2^_*p*_ = 0.25; drug x phenotype, *F* < 1).

#### Summary

Chronic quinpirole treatment increased functional checking in goal-trackers, during and even after treatment (Fig. 2c). Chronic quinpirole did not affect dysfunctional checking, which was elevated in both goal-trackers (Fig. 2g) and sign-trackers (Fig. 2h) relative to non-autoshaped controls. The dose of quinpirole was pharmacologically active, producing non-specific effects on active and inactive lever pressing (Supplementary Figure 2). Furthermore, chronic quinpirole reduced accuracy in lever choice when the cue light was off, particularly in autoshaped rats (Fig. 3g). This may reflect quinpirole’s effects on working memory or attention, which would reduce the ability of rats to direct responding to the correct lever, and consequently increase functional checking.

### Effects of reward omission on functional and dysfunctional checking

#### Functional checking

**Fig. 4:**
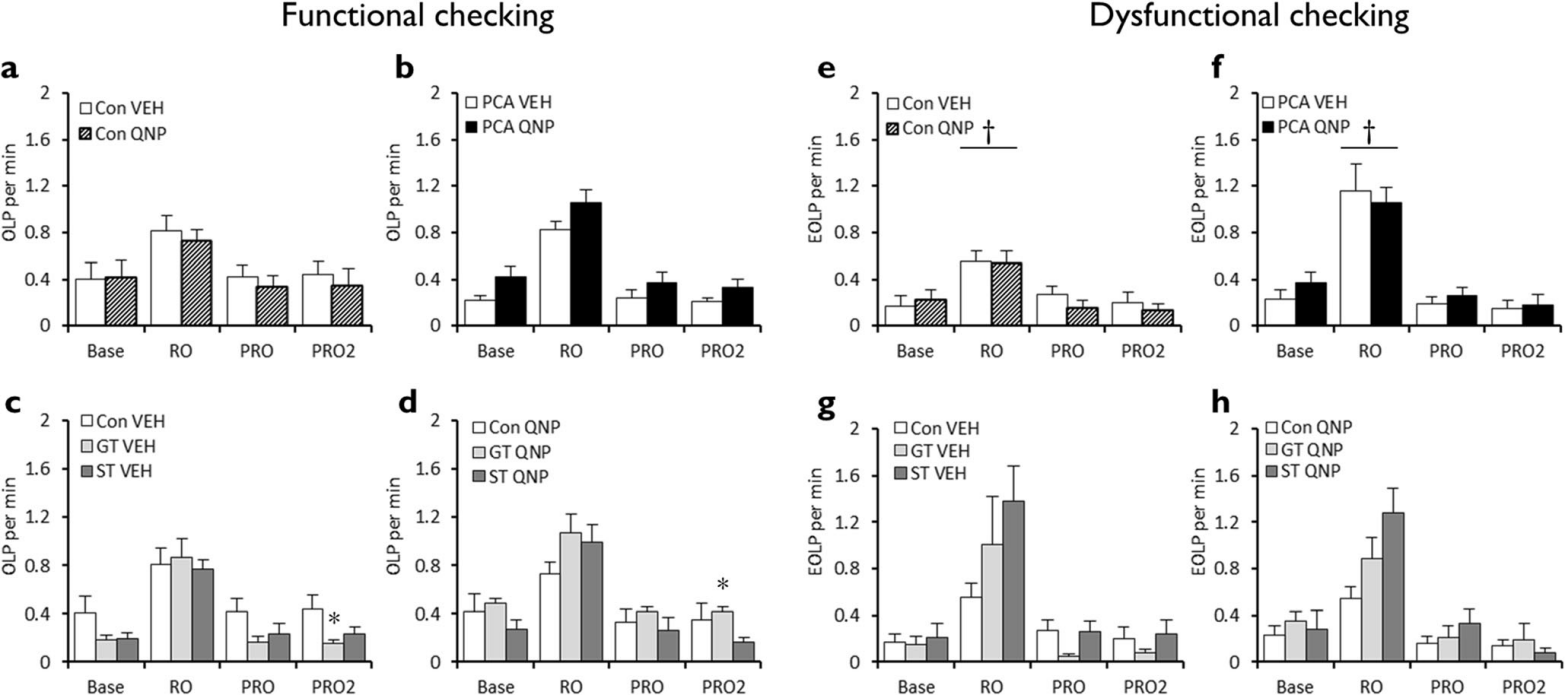
Effects of prior chronic quinpirole treatment and reward omission on functional and dysfunctional checking on the ORT. Reward omission increased functional checking in both non-autoshaped control (**a**) and autoshaped (**b**) rats, with checking levels rapidly returning to baseline when reward was re-introduced. There were no differences between goal-trackers (**c**) and sign-trackers (**d**) in their functional checking during reward omission. Control rats showed an increase in dysfunctional checking under reward omission (**e**) that was further elevated in rats that had undergone autoshaping (**f**). Across prior vehicle-treated (**g**) and prior quinpirole-treated (**h**) groups, sign-trackers tended to show higher levels of dysfunctional checking than goal-trackers and controls. ‘Base’ is baseline responding, RO the reward omission session, PRO and PRO2 the two post-reward omission sessions. Con, control group; PCA, autoshaped group; QNP, quinpirole-treated group; VEH, vehicle-treated group; GT, goal-trackers; ST, sign-trackers. Data are means ± s.e.m. Group sizes: Con VEH, *n* = 12; Con QNP, *n* = 12; PCA VEH, *n* = 12, PCA QNP, *n* = 12; GT VEH, *n* = 5; GT QNP, *n* = 6; ST VEH, *n* = 6; ST QNP, *n* = 5. Differences of *p* < .05 between non-autoshaped controls and autoshaped groups indicated by †

Reward omission increased functional OLPs in all animals, with a rapid return to baseline levels of checking when reward was reintroduced (block, *F*_(2.65,117)_ = 96.5, *p* < .001, *η*^2^_*p*_ = 0.69). Quinpirole treatment history did not affect the number of OLPs made during reward omission (drug, *F* < 1; block x drug, *F* < 1), but autoshaped rats made more OLPs than non-autoshaped controls during reward omission (Figs. 4a, b; block x pretraining, *F*_(2.65,117)_ = 6.76, *p* < .001, *η*^2^_*p*_ = 0.13). There are no overall differences in the OLPs of goal-trackers and sign-trackers (Fig. 4c, d) during or after reward omission (phenotype, *F* < 1; block x phenotype, *F*_(1,18)_ = 1.30, *p* = .27).

#### Dysfunctional checking

Reward omission increased dysfunctional eOLPs in both non-autoshaped controls (Fig. 4e) and autoshaped rats (Fig. 4f; block, *F*_(1.44,63.2)_ = 48.8, *p* < .001, *η*^2^_*p*_ = 0.53). The increase in dysfunctional eOLPs produced by reward omission was greater in autoshaped rats than non-autoshaped controls (pretraining, *F*_(1,44)_ = 6.13, *p* = .017, *η*^2^_*p*_ = 0.12; block x pretraining, *F*_(1.44,63.2)_ = 9.06, *p* = .001, *η*^2^_*p*_ = 0.17). Quinpirole treatment history did not affect eOLPs made during reward omission (Fig. 4e, f; drug, *F* < 1; block x drug, *F* < 1). Goal-trackers and sign-trackers both increased numbers of eOLPs made during reward omission, regardless of whether they had received vehicle (Fig. 4g) or quinpirole (Fig. 4h) previously (phenotype, *F* < 1; block x phenotype, *F*_(1.24,22.3)_ = 1.16, *p* = .31).

#### Summary

Reward omission acutely increased both functional and dysfunctional checking (Fig. 4) and selectively reduced active lever pressing (see Supplementary Materials). The effects of reward omission were transient, with responding rapidly returning to baseline when reward was returned. Prior quinpirole treatment did not affect checking during reward omission, and both sign-trackers and goal-trackers showed increased functional checking when reward was omitted.

### Effects of uncertainty on functional and dysfunctional checking

#### Functional checking

**Fig. 5:**
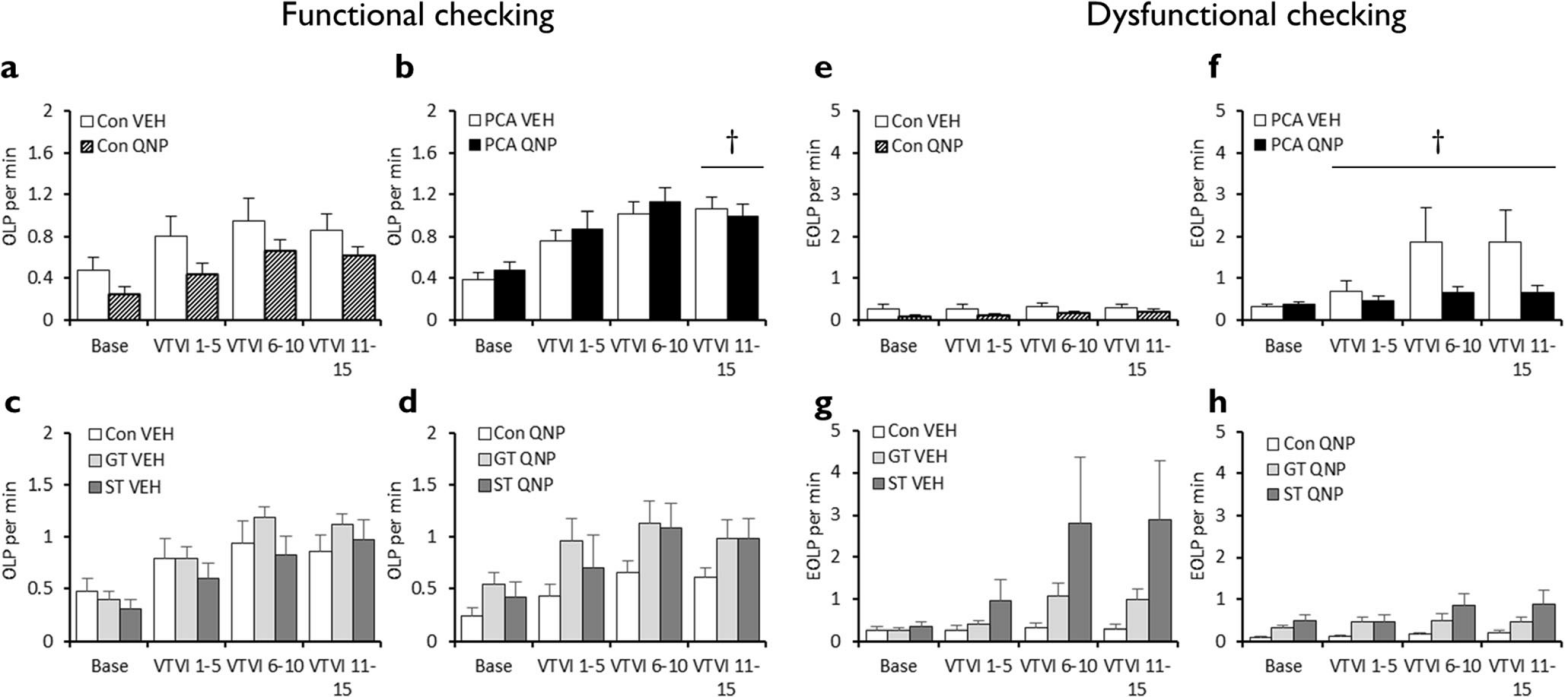
Effects of prior chronic quinpirole treatment and uncertainty on functional and dysfunctional checking on the ORT**.** The introduction of uncertainty increased functional checking in both control (**a**) and autoshaped rats (**b**) with no overall effects of prior quinpirole treatment, and no differences between goal-trackers (**c**) and sign-trackers (**d**). Uncertainty did not affect dysfunctional checking in non-autoshaped control rats (**e**) but increased dysfunctional checking in autoshaped rats (**f**). Further analyses revealed that dysfunctional checking was markedly increased in vehicle-treated sign-tracker rats (**g**) and that prior treatment with quinpirole normalised this elevated dysfunctional checking (**h**). ‘Base’ is baseline responding, VTVI1–5, VTVI6–10 and VTVI11–15 the first, second and third blocks of the uncertainty (variable time/variable interval) sessions. Con, control group; PCA, autoshaped group; QNP, quinpirole-treated group; VEH, vehicle-treated group; GT, goal-trackers; ST, sign-trackers. Data are means ± s.e.m. Group sizes: Con VEH, *n* = 12; Con QNP, *n* = 12; PCA VEH, *n* = 12, PCA QNP, *n* = 12; GT VEH, *n* = 5; GT QNP, *n* = 6; ST VEH, *n* = 6; ST QNP, *n* = 5. Differences of *p* < .05 between non-autoshaped controls and autoshaped groups indicated by †

The introduction of uncertainty increased functional OLPs for both non-autoshaped controls (Fig. 5a) and autoshaped rats (Fig. 5b) across the three blocks of testing (block, *F*_(3,132)_ = 68.2, *p* < .001, *η*^2^_*p*_ = 0.61). Quinpirole treatment did not affect OLPs made under uncertainty (drug, *F* < 1; block x drug, *F* < 1) though autoshaped rats made more OLPs than non-autoshaped controls (block x pretraining, *F*_(3,132)_ = 2.75, *p* = .046, *η*^2^_*p*_ = 0.06). There were no differences between goal-trackers and sign-trackers in the number of functional OLPs made during uncertainty, regardless of whether they had received vehicle (Fig. 5c) or quinpirole (Fig. 5d) previously (phenotype, *F* < 1; block x phenotype, *F* < 1).

#### Dysfunctional checking

Uncertainty did not affect the number of dysfunctional eOLPs made by non-autoshaped controls (Fig. 5e) but elevated these in autoshaped rats (Fig. 5f; pretraining, *F*_(1,44)_ = 7.29, *p* = .01, *η*^2^_*p*_ = 0.14; block x pretraining, *F*_(1.13,49.7)_ = 4.94, *p* = .027, *η*^2^_*p*_ = 0.10). Quinpirole treatment history did not affect the number of eOLPs made under uncertainty when both autoshaped and non-autoshaped rats were analysed (Fig. 5e, f; drug, *F*_(1,44)_ = 2.70, *p* = .11; block x drug, *F*_(1.13,49.7)_ = 2.66, *p* = 0.11), but there was a trend towards an interaction between quinpirole treatment and prior autoshaping experience (Fig. 5f; block x drug x pretraining, *F*_(1.13,49.7)_ = 3.08, *p* = .081, *η*^2^_*p*_ = 0.066). Within the autoshaped group, there was a trend towards both goal-trackers and sign-trackers being affected by prior quinpirole treatment (Fig. 5h; block x drug, *F*_(1.12,20.2)_ = 2.91, *p* = .10, *η*^2^_*p*_ = 0.14). While vehicle-treated rats (Fig. 5g) escalated their dysfunctional checking during uncertainty (sessions 11–15 differed from baseline and sessions 1–5, *p*’s < .03), rats that had previously received quinpirole treatment (Fig. 5h) did not (all *p*’s > .99).

Although the omnibus ANOVA did not show any overall differences in the dysfunctional checking of goal-trackers and sign-trackers during the uncertainty sessions (phenotype, *F*_(1,18)_ = 1.67, *p* = .21; block x phenotype, *F*_(1.12,20.2)_ = 1.62, *p* = .22; block x drug x phenotype, *F* < 1), based on our *a priori* hypothesis that goal-trackers and sign-trackers would behave differently based on prior quinpirole treatment, we examined this further with Šidák-corrected pairwise comparisons (Cardinal and Aitken 2006). These showed differences in dysfunctional checking between goal-trackers and sign-trackers treated with vehicle and quinpirole. Quinpirole-treated rats did not alter their levels of dysfunctional checking across the uncertainty sessions, regardless of whether they were goal-trackers or sign-trackers (all *p*’s > .97). For the vehicle-treated rats (Fig. 5g), goal-trackers did not elevate their responding beyond baseline levels during the uncertainty sessions (all *p*’s > .90) but sign-trackers increased their dysfunctional checking progressively across the uncertainty sessions (compared with baseline, sessions 1–5 trended towards higher dysfunctional checking, *p* = .064, while sessions 6–15 had greater dysfunctional checking with *p*’s < .039).

#### Summary

Uncertainty increased functional checking in all animals (Fig. 5a–d), and dysfunctional checking was further elevated in autoshaped rats (Fig. 5f). Sign-trackers showed the greatest escalation in dysfunctional checking under uncertainty and, perhaps counterintuitively, prior chronic treatment with quinpirole normalised this.

## Discussion

Checking, elicited by quinpirole sensitization or uncertainty in the observing response task, was increased by prior appetitive pavlovian conditioning. Whereas goal-trackers increased functional checking following chronic quinpirole treatment, sign-trackers did not. By contrast, dysfunctional checking was increased by introducing uncertainty to the observing response task, whether through the omission of expected reward or alteration of both the reinforcement schedule and the predictability of switching between the active and inactive levers. Under these conditions, autoshaped rats showed greater levels of dysfunctional checking, with sign-trackers showing particularly elevated levels. Quinpirole did not affect the elevated levels of dysfunctional checking made by autoshaped rats during reward omission and appeared to normalise the high levels of dysfunctional checking made by sign-trackers under uncertainty conditions. Thus, the elevated dysfunctional checking shown by autoshaped rats, particularly sign-trackers (present data and Vousden et al. 2020) under conditions of uncertainty, may provide a new model of checking in OCD.

Although the study was designed to maximise effects on checking by autoshaping, uncertainty and quinpirole sensitization, these factors did not interact additively. Quinpirole increased levels of functional checking in previously autoshaped rats with a moderate effect size, potentially because of its large impairing effect on discrimination between the active and inactive levers in the absence of the identifying cue light. Chronic quinpirole failed to produce dysfunctional checking itself and in fact normalised the elevated dysfunctional checking made under conditions of uncertainty. This study thus helps to define conditions under which checking behaviour, as an adaptive form of information-seeking, may become excessive and/or dysfunctional, corresponding to the compulsive checking exhibited in OCD, and informs the use of quinpirole sensitization in animal analogues of OCD. Dysfunctional checking is more relevant to the debilitating and dysfunctional symptoms of OCD, particularly when excessive. We propose that autoshaped rats, and particularly sign-trackers, which showed increases of large effect size in dysfunctional checking under uncertainty, are a more appropriate model for OCD compulsive checking symptoms than quinpirole sensitization, which promoted more functional than dysfunctional checking.

### Neurobehavioural mechanisms of functional checking

Functional checking provides information to guide instrumental reward-seeking in both rat and human ORT studies (d’Angelo et al. 2017; Eagle et al. 2014; Morein-Zamir et al. 2018). Information-seeking is particularly appropriate under conditions of uncertainty, for example, produced by reward omission or unpredictable reinforcement schedules. Functional checking in the ORT increases during conditions of uncertainty, potentially triggered as part of a security motivation system (Szechtman and Woody 2004) responsive to stressful or uncertain conditions; there is a strong link between uncertainty and information-seeking (Anselme et al. 2013). Uncertainty may also result from impaired perception or working memory associated with food-seeking, as may occur following nucleus accumbens lesions or inactivation (d’Angelo et al. 2017; Floresco et al. 2018). This form of uncertainty may have precipitated the present effects of quinpirole, which included not only reductions in instrumental responding, but also failures to discriminate between active and inactive levers in the absence of the cue light, thus producing increases in functional checking. Consequently, functional checking escalation itself may not be a critical component of a more pathological OCD-like model, although it is possible that when functional checking is driven to excess by other factors, it may become maladaptive.

An alternative hypothesis is that functional checking produces a conditioned reinforcer in the form of a reward-predictive CS, which not only provides information, but also has affective value (Dinsmoor 1983). However, some evidence against this perspective arises from the effects of nucleus accumbens lesions, which reduce control over choice behaviour by conditioned reinforcers (Parkinson et al. 1999), and yet lead to increases in functional checking (d’Angelo et al. 2017).

Autoshaped rats showed an increase of large effect size in functional checking following quinpirole sensitization and were also less able to determine which lever was currently reinforced during and after chronic quinpirole treatment, supporting the relationship between functional checking and information-seeking. We therefore conclude that functional checking escalation following quinpirole is an adaptive response, directly linked to the extent to which quinpirole impairs instrumental responding in the absence of appropriate task information. However, while both goal-trackers and sign-trackers were equally impaired under quinpirole in determining which lever was active in the absence of cue light information, it was selectively goal-trackers that showed elevated functional checking following chronic quinpirole treatment. Goal-trackers are thought to be more sensitive to the influence of contextual cues than sign-trackers (Morrow et al. 2011; Pitchers et al. 2017; Saunders et al. 2014). It is possible that goal-trackers were more sensitive to the differences in internal state before, during and after chronic quinpirole treatment, increasing their requirement for information-seeking and therefore functional checking. The elevated functional checking we observed here is consistent with our earlier report on the effects of quinpirole, which however also showed inconsistent increases in dysfunctional checking, perhaps due to the relative distribution of sign-trackers and goal-trackers in these two studies. The current data are most directly comparable with the low-checker group from our previous study (Eagle et al. 2014).

### Neurobehavioural mechanisms of dysfunctional checking

Dysfunctional checking does not provide information and may become maladaptive when excessive. Sign-trackers exhibited clear patterns of checking-response escalation during uncertainty, with a bias towards increased dysfunctional checking.

There are several neurobehavioural mechanisms that could underlie increased dysfunctional checking. Sign-trackers, by definition, exhibit enhanced approach responses to pavlovian CSs. The checking lever functions as a CS+, as well as being an instrumental response during the ORT, producing information about future food reinforcement. Consequently, the dysfunctional checking of sign-trackers could be considered as pavlovian-to-instrumental transfer (PIT), by which CSs modulate instrumental responses (Cartoni et al. 2016). It has recently been postulated that OCD patients fail to integrate goal-directed and habitual control as a consequence of exaggerated PIT (Bradfield et al. 2017). However, against this interpretation, there is no corresponding increase in instrumental responding for food on the active or inactive levers, which would be expected of both general and specific forms of PIT.

An alternative explanation might be recruitment of habitual control (Bradfield et al. 2017; Watson and de Wit 2018) over the checking response. This explanation depends on the instrumental nature of checking and assumes that extended training results in a habitual tendency to press the checking lever and that this tendency is greater in sign-trackers. It is important to note that although the checking lever serves as a pavlovian stimulus during autoshaping, it is an instrumental response manipulandum during the ORT. If sign-trackers were simply more engaged with the lever as a pavlovian stimulus, then it would be predicted that they would show non-specific increases in both functional and dysfunctional checking across all conditions, rather than a selective increase in dysfunctional checking, particularly under conditions of uncertainty. An account based upon sign-trackers rapidly progressing to habitual responding may be supported by evidence that autoshaped rats respond more for the CS+ when it is a conditioned reinforcer, with such responding gaining habitual qualities (Parkinson et al. 2005). Sign-trackers also exhibit greater control by conditioned reinforcers of instrumental responding (Yager and Robinson 2013) and do not reduce responding for CSs following outcome devaluation with lithium chloride (Morrison et al. 2015; Nasser et al. 2015). However, further experiments involving devaluation of the informational value of the light cue in the ORT would be required to explicitly test this hypothesis.

### The importance of dopamine for functional and dysfunctional checking

Though the administration of quinpirole in the current study was systemic, we speculate that the effects of quinpirole on checking behaviour are mediated by its action in the nucleus accumbens. Nucleus accumbens dopamine function has a clear role in OCD-relevant checking behaviour, sign-tracker/goal-tracker trait expression and response to reward uncertainty (Anselme et al. 2013; Ballester González et al. 2015; Eagle et al. 2014; Flagel and Robinson 2017; Flagel et al. 2007). For example, both quinpirole sensitization and nucleus accumbens lesions increase checking behaviour in a manner superficially comparable with OCD compulsive checking (Ballester González et al. 2015; d’Angelo et al. 2017; Dvorkin et al. 2010; Eagle et al. 2014). Rats make more visits to a home base in open-field testing (Dvorkin et al. 2010) and check more in the ORT. During quinpirole sensitization, there are reports of reduced dopamine in the nucleus accumbens core (de Haas et al. 2011; Escobar et al. 2017), increased D2-receptor post-synaptic sensitivity (Escobar et al. 2017), increased D2-receptor binding and decreased glucose utilisation (Culver et al. 2008). Quinpirole sensitization effects on checking and other aspects of task performance, at least during the treatment period, likely result from reduced dopamine function in the nucleus accumbens core. This hypothesis should receive attention in future research using the ORT.

Dopamine signalling in the nucleus accumbens core has also been proposed as critical to the attribution of incentive salience properties during both acquisition and maintenance of sign-tracking (Flagel and Robinson 2017). Sign-trackers develop clear CS-evoked dopamine release in the nucleus accumbens, whereas goal-trackers do not, instead displaying a lesser US-evoked response (Flagel et al. 2011). Acquisition and maintenance of sign-tracking are dependent on dopamine receptor function, with both being impaired following treatment with α-flupenthixol (Flagel et al. 2011) and mesolimbic dopamine depletion (Parkinson et al. 2002). However, the precise mechanism through which sign-tracking is modulated remains unclear. It has been reported that rats inbred for sign-tracking phenotype are more sensitive to quinpirole, with some evidence that this is also the case for outbred strains (Flagel et al. 2010). However, it has also been shown that with extended training, sign-tracking becomes less dependent on dopaminergic signalling (Clark et al. 2013). Therefore, the failure of quinpirole to interact additively to enhance the dysfunctional checking shown by sign-trackers, particularly under conditions of uncertainty, may be related to the extent of autoshaping training experienced by the animals in this experiment.

There is a strong link between dopamine function and reward uncertainty (Cocker et al. 2012; Fiorillo et al. 2003; Floresco 2013). Of particular relevance to this study, sign-tracking escalates over time in the face of uncertain reward during autoshaping training (Anselme 2015), with both uncertainty and sign-tracking linked with phasic peaks in dopamine release (Fiorillo et al. 2003; Flagel et al. 2011; Mascia et al. 2018). Therefore, it is likely that uncertainty and sign-tracking influences on checking behaviour are linked with increased dopamine function.

### Implications and limitations for models of OCD

This study provides evidence for the importance of previous autoshaping experience and dopaminergic manipulation on the development and escalation of OCD-like checking. Although quinpirole sensitization implicates dopamine in the escalation of checking, chronic quinpirole produced elevation only of functional checking, with only a trend towards a small-moderate effect. Dysfunctional checking was more evident in sign-tracking rats, particularly under conditions of uncertainty where there was an increase in dysfunctional checking with large effect size, and this was ameliorated by quinpirole treatment. It is currently unclear whether sign-tracking causes dysfunctional checking or if it is a marker of susceptibility of a motivational system prone to aberrant and maladaptive habit development.

The unpredicted failure of sign-tracking to synergize with the effects of chronic quinpirole to produce dysfunctional checking as a consequence of the presumed elevations in D_2_ receptor sensitivity in such rats may have been due to the use of outbred, rather than inbred bHR strains, where the latter changes have been more extensively characterised. The adaptive/maladaptive nature of checking has been probed further with the introduction of aversive consequences of failure to check (Vousden et al. 2020), given that an important component of OCD models is compulsive behaviour maintained despite aversive consequences. Additionally, the parallel human analogue of the ORT (Morein-Zamir et al. 2018) gives the task potential translational value. Furthermore, given the recent finding that sign-tracking correlates with questionnaire measures of compulsive behaviours (Albertella et al. 2019), an evaluation of the relationship between sign-tracking and checking behaviour in humans would be invaluable.

Sign-tracking and goal-tracking phenotypes differentially affected functional and dysfunctional checking. Sign-trackers showed more evidence of the latter, particularly under conditions of uncertainty. By contrast, their response to quinpirole was reduced compared with goal-trackers, who exhibited more functional checking before and after chronic quinpirole treatment. These findings have important implications for translational models of checking behaviour in conditions such as OCD.

## Electronic supplementary material

ESM 1 (PNG 2706 kb)

High Resolution Image (TIF 471 kb)

ESM 2 (PNG 1380 kb)

High Resolution Image (TIF 459 kb)

ESM 3 (DOCX 17 kb)
