# Supplementary figures and images for: Dissociable dopaminergic and pavlovian influences in goal-trackers and sign-trackers on a model of compulsive checking in OCD
Source: Psychopharmacology (Berl). 2020 Sep 4;237(12):3569–81. doi: 10.1007/s00213-020-05636-3 (PMC7683452; doi:10.1007/s00213-020-05636-3)

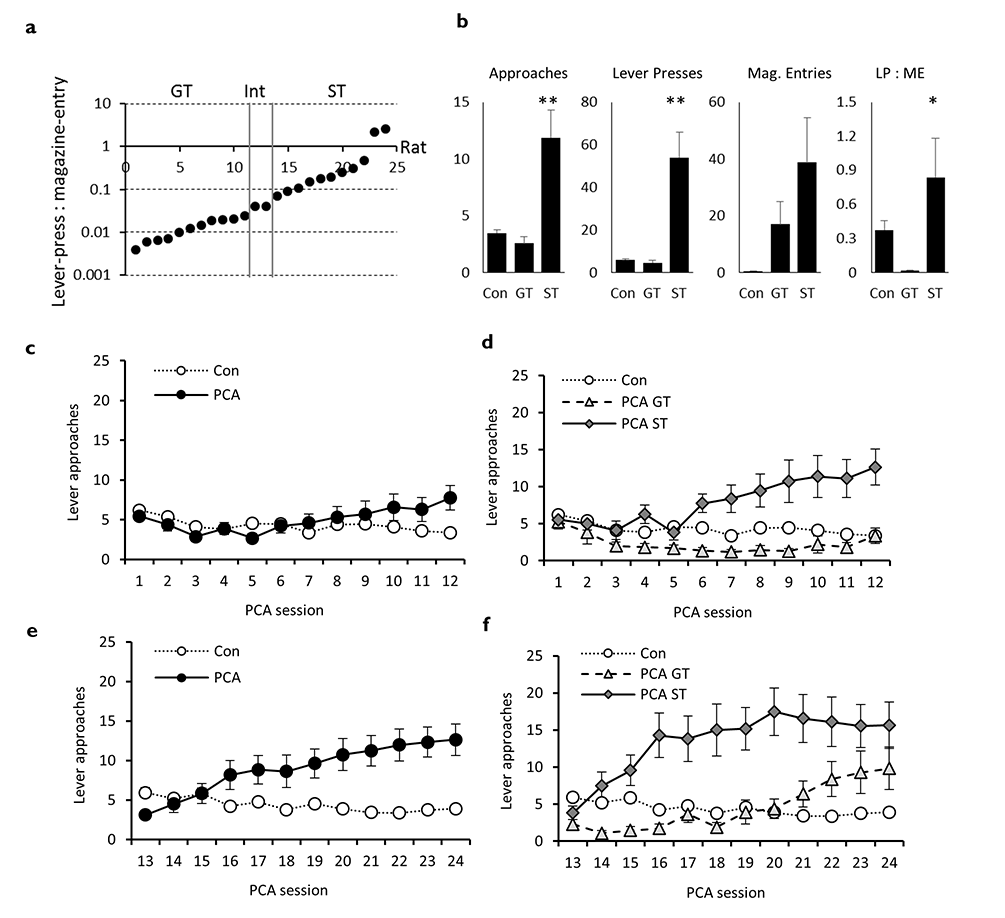

Supplement: Supplementary file 1 — (PNG 2706 kb) [file 213_2020_5636_Fig6_ESM.png]

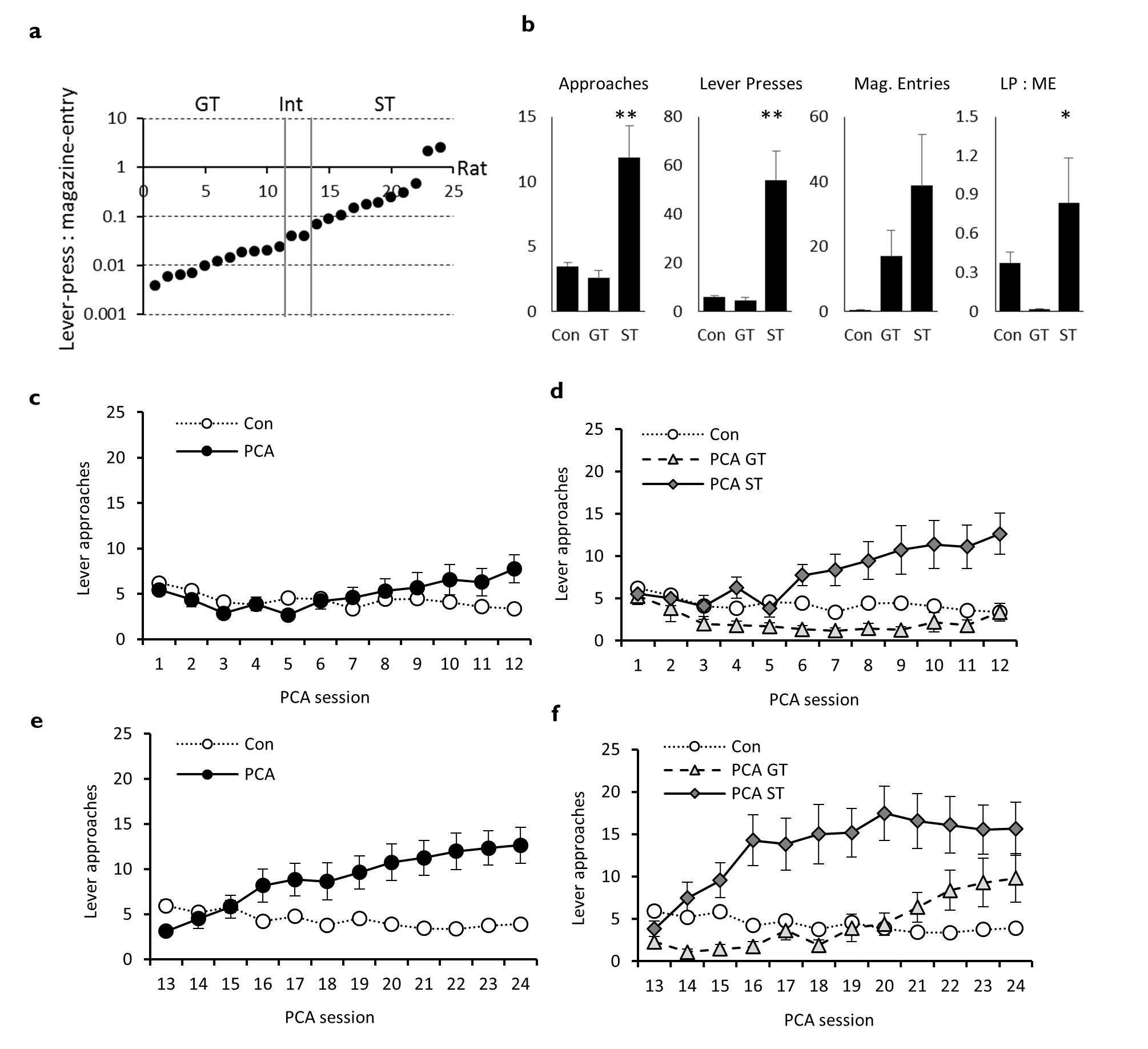

Supplement: Supplementary file 2 — High Resolution Image (TIF 471 kb) [file 213_2020_5636_MOESM1_ESM.tif]

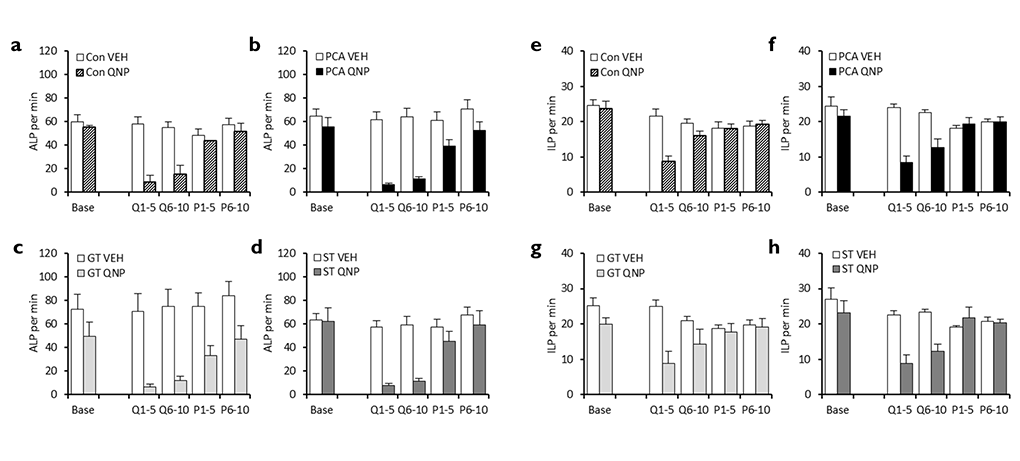

Supplement: Supplementary file 3 — (PNG 1380 kb) [file 213_2020_5636_Fig7_ESM.png]

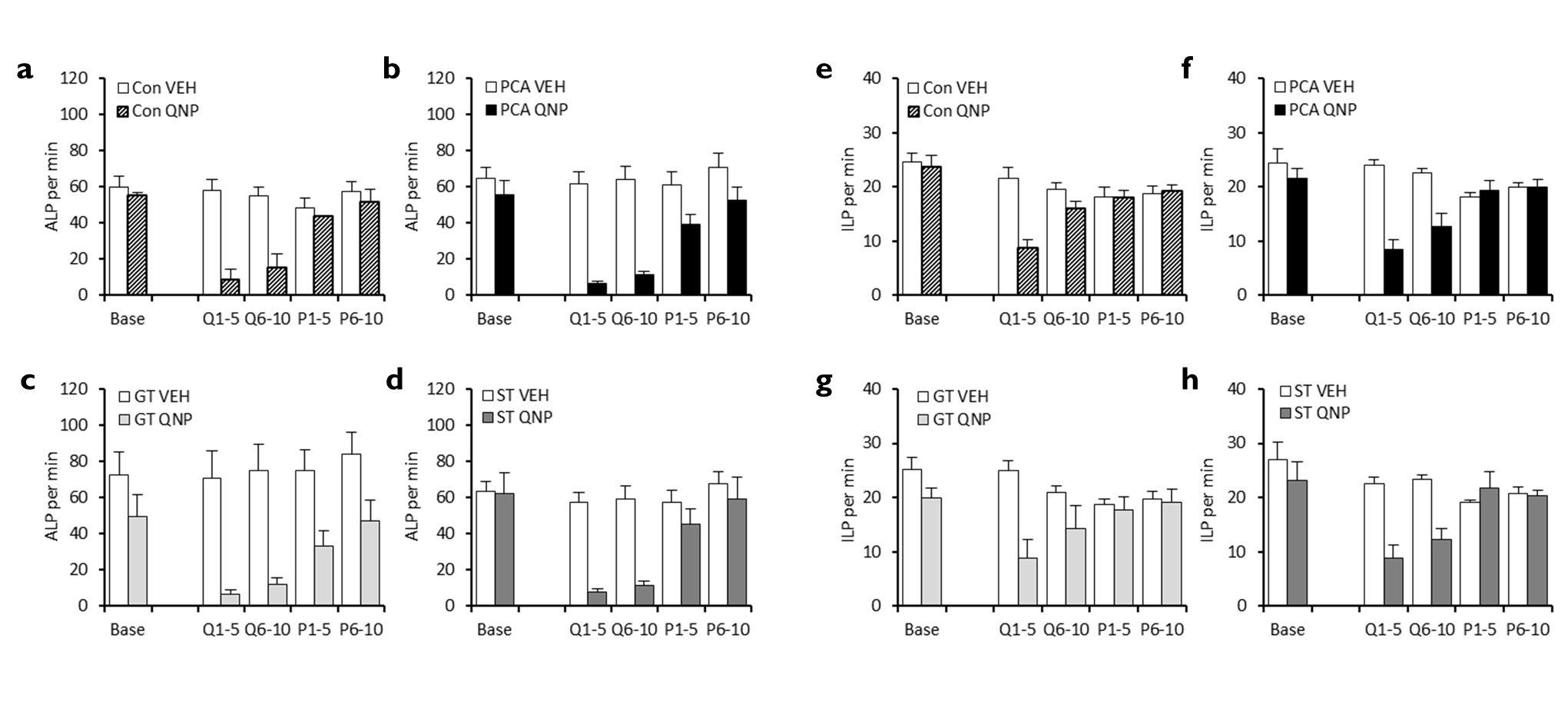

Supplement: Supplementary file 4 — High Resolution Image (TIF 459 kb) [file 213_2020_5636_MOESM2_ESM.tif]
