# Supplementary material for: Dissociable dopaminergic and pavlovian influences in goal-trackers and sign-trackers on a model of compulsive checking in OCD
Source: Psychopharmacology (Berl). 2020 Sep 4;237(12):3569–81. doi: 10.1007/s00213-020-05636-3 (PMC7683452; doi:10.1007/s00213-020-05636-3)
Supplement: Supplementary file 5 — (DOCX 17 kb) [file 213_2020_5636_MOESM3_ESM.docx]

**SUPPLEMENTARY RESULTS**

## Quinpirole and PCA effects on functional (OLP) and dysfunctional (eOLP) checking

## Pre-drug treatment (baseline) measures. There were no differences in performance between the (prospective) quinpirole (QNP) and vehicle (VEH) groups (leftmost ‘Base’ bars in Figures 2 and 3 and Supplementary Figure 2). There were no differences in observing lever presses (OLPs) [*F*<1], extra observing lever presses (eOLPs) [*F*_(1,47)_=1.43, *p*=.24], discrimination between the active and inactive levers when the light was on (‘light-on discrimination’) [*F*_(1,47)_=3.48, *p*=.07] or when the light was off (‘light-off discrimination’) [*F*_(1,47)_=1.18, *p*=.28].

There were no differences between autoshaped and control groups on OLPs [*F*<1], eOLPs [*F*_(1,47)_=1.50, *p*=.23], light-on discrimination [*F*<1] or light-off discrimination [*F*_(1,47)_=1.54, *p*=.22]. Therefore, the groups were well-matched prior to the start of the pharmacological manipulations.

**Effects of Reward Omission (RO) on functional and dysfunctional checking**

**Pre-reward omission manipulation (baseline) measures.** There were no baseline differences between previously vehicle-treated and quinpirole-treated rats (leftmost ‘Base’ bars in **Supplementary Figure 3**) in OLPs [*F*<1], eOLPs [*F*_(1,47)_=1.53, *p*=.22], or light-on [*F*<1] or light-off discrimination [*F*_(1,47)_=2.04, *p*=.16].

There were no differences between control and autoshaped animals on OLPs [*F*<1], eOLPs [*F*_(1,47)_=1.66, *p*=.20], or light-on [*F*<1] or light-off discrimination [*F*<1]. Therefore, the previous treatment with chronic quinpirole did not affect baseline responding in a manner that would complicate the subsequent interpretation of the reward omission sessions.

**Active lever pressing.** Active lever pressing (data not shown) decreased under reward omission but rapidly returned to baseline when reward was re-introduced [*F*_(1.33,58.5)_=60.2, *p*<.001, η^2^=0.58] for all rats [Pretraining: *F*_(1,44)_=1.62, *p*=.21; Block*Pretraining: *F*_(1.33,58.5)­_=1.46, *p*=.24; Drug: *F*_(1,44)_=2.84, *p*=.099; Block*Drug: *F*<1]. Further analyses revealed no differences in active lever pressing between sign-trackers and goal-trackers [*F*<1].

**Inactive lever pressing.** Inactive lever pressing did not decrease any further under reward omission for any experimental groups [Block: *F<*1; Drug: *F*<1; Block*Drug: *F*<1; Pretraining: *F*<1; Block*Pretraining, *F*<1]. Further analyses also confirmed no differences between sign-trackers and goal-trackers [Phenotype: *F*_(1,18)_=1.47, *p*=.24; Block*Phenotype: *F*<1].

**Discrimination between active and inactive levers.** Light-on discrimination (data not shown) did not vary based on prior quinpirole treatment [*F*<1], autoshaping history [*F*<1] or sign-tracking or goal-tracking phenotype [Phenotype: *F*<1; Block*Phenotype: *F<*1].

Light-off discrimination was reduced during reward omission compared to subsequently rewarded sessions [*F*_(1.86,81.9)_=31.4, *p*<.001, η^2^=0.42], similarly for all animals [Drug: *F*<1; Block*Drug: *F*<1; Pretraining: *F*<1; Block*Pretraining: *F*<1] with no differences between sign-trackers and goal-trackers [Phenotype: *F*<1; Block*Phenotype: *F*<1].

**Effects of uncertainty on functional and dysfunctional checking**

**Pre-uncertainty (baseline) measures.** There were no baseline differences between previously vehicle-treated and quinpirole-treated rats (leftmost ‘Base’ bars in **Figure 4**) in OLPs [*F*<1], eOLPs [*F*< 1], light-on [F<1] or light-off [F_(1,47)_=2.04, p=.16] discrimination.

There were also no differences between control and autoshaped animals in OLPs [*F*<1], eOLPs [*F*_<_1], light-on [*F*<1] or light-off discrimination [*F*<1]. Therefore, the previous treatment with chronic quinpirole did not affect baseline responding in a manner that would complicate the subsequent interpretation of the uncertainty sessions.

**Active lever pressing.** Active lever pressing (data not shown) decreased under uncertainty [*F*_(1.30,57.3)_=48.9, *p*<.001, η^2^=0.53], as would be expected from a less predictable reinforcement schedule, for all rats [Pretraining: *F*_(1,44)_=2.00, *p*=.16; Block*Pretraining: *F<*1]. This was not affected by prior quinpirole treatment [Drug: *F<*1; Block*Drug: *F*<1] or sign-tracking or goal-tracking phenotype [*F*<1].

**Inactive lever pressing.** Rats made a greater number of errors and therefore inactive lever pressing increased under uncertainty conditions (data not shown) [Block: *F*_(1.54,67.8)_=26.7, *p*<.001, η^2^=0.38]. Autoshaped rats made more inactive lever presses [Block*Pretraining: *F*_(1.54,67.8)_=4.02, *p*<.05, η^2^=0.084] and prior quinpirole exposure produced different effects on autoshaped and control rats [Block*Drug*Pretraining: *F*_(1.54,67.8)_=4.06, *p*<.05, η^2^=0.085]. Controls did not differ in inactive lever pressing under uncertainty irrespective of drug treatment [all *p*’s>.081], but prior quinpirole reduced inactive lever pressing in the final block of uncertainty for autoshaped rats [*p*=.038] for both sign-trackers and goal-trackers [Phenotype: *F<*1; Block*Phenotype: *F*<1].

**Discrimination between active and inactive levers.** Light-on discrimination (data not shown) was better overall in control than autoshaped rats [*F*_(1,44)_=5.57, *p*=.023, η^2^=0.11] irrespective of prior quinpirole treatment [*F*_(1,44)_=1.43, *p*=.24]. Goal-trackers showed better light-on discrimination than sign-trackers [Phenotype: *F*_(1,18)­=_6.04, *p*=.024, η^2^=0.25] and previously quinpirole-treated rats showed a trend towards better discrimination [Drug: *F*_(1,18)_=4.31, *p*=.052, η^2^ =0.19]. There was, however, no interaction between prior quinpirole treatment and sign-tracking or goal-tracking phenotype [Drug*Phenotype: *F*_(1,18)_=1.52, *p*=.23].

There was no effect of prior autoshaping or prior quinpirole treatment on light-off discrimination [Pretraining: *F*<1; Drug: *F*<1], with no differences between sign-trackers and goal-trackers [Phenotype: *F*<1; Drug: *F<*1].

**SUPPLEMENTARY FIGURE LEGENDS**

**Figure S1.** **Pavlovian conditioned approach (autoshaping). (A)** Ratio of lever press:magazine entry during 30 x 10-second lever presentations for all rats. Lines demark allocation to the goal-tracker (GT), intermediate (Int) and sign-tracker (ST) groups. **(B)** Autoshaping performance at the end of training (means of Sessions 11-12). **(C,D)** Approaches to the lever during initial training and **(E, F)** during retraining.

**Figure S2. Chronic quinpirole reduced instrumental responding on the ORT. (A)** Active lever presses were reduced by chronic quinpirole treatment in both autoshaped (PCA) and control animals (top panels). Goal-trackers and and sign-trackers showed similar reductions in lever pressing, though in goal-trackers this persisted into the early post-treatment period (bottom panels). **(B)** Inactive lever pressing was also reduced by quinpirole treatment in both control and autoshaped animals (top panels), with a more rapid recovery in levels of responding in goal-trackers compared to sign-trackers (bottom panels). ‘Base’ is baseline responding, Q1-5 and Q6-10 the first and second blocks of chronic VEH/QNP treatment, P1-5 and P6-10 the two post-treatment session blocks. Con, control group; PCA, autoshaped group; QNP, quinpirole-treated group; VEH, vehicle treated group; GT, goal-trackers, ST, sign-trackers. Data are means ± s.e.m. Group sizes:Con VEH, n=12; Con QNP, n=12; PCA VEH, n=12, PCA QNP, n=12; GT VEH, n=5; GT QNP, n=6; ST VEH, n=6; ST QNP, n=5.
